# Haploidentical hematopoietic stem cell transplantation as a first-line treatment for paediatric severe aplastic anemia: a single-center research

**DOI:** 10.7150/ijms.94012

**Published:** 2024-04-15

**Authors:** Meng Yuan, Chenguang Jia, Jie Ma, Man Zhang, Guanghua Zhu, Bin Wang, Jie Zheng, Maoquan Qin, Runhui Wu, Sidan Li

**Affiliations:** 1 Hematology Center, Beijing Key Laboratory of Pediatric Hematology Oncology; National Key Discipline of Pediatrics (Capital Medical University); Key Laboratory of Major Diseases in Children, Ministry of Education; Beijing Children's Hospital, Capital Medical University, National Center for Children's Health, Beijing, China; 2 Department of Medical Oncology, National Cancer Center/National Clinical Research Center for Cancer/Cancer Hospital, Chinese Academy of Medical Sciences and Peking Union Medical College, Beijing, China.

**Keywords:** aplastic anemia, hematopoietic stem cell transplantation, immunosuppressive therapy, children

## Abstract

MRD-HSCT is the first-line therapy for children with SAA, while it is not easy to find a compatible donor due to the Chinese one-child policy. IST has a high recurrence rate, a risk of clonal transformation. Thus, Haplo-HSCT, as a first-line treatment, has gradually attracted clinicians' attention. To evaluate the efficacy of Haplo-HSCT in children with SAA, we performed a retrospective study (2006.06-2021.01) of 210 patients with AA who received HSCT or IST in Beijing Children's Hospital. The OS and FFS rates were analyzed to evaluate the efficacy of Haplo-HSCT and IST. We found that from 2006 to 2021, 3- and 5-year cumulative survival rates were both 85.3% in the first-line Haplo group, 98.1% and 96.8% in the first-line IST group, both 85.7% in the ATG group (P = 0.866), both 100% in the ATG + TPO group (P = 0.016), and 99.1% and 97.2% in the ATG + eltrombopag group (P = 0.056). 3- and 5-year cumulative FFS rates were both 85.3% in the first-line Haplo-HSCT group and 67.5% and 66.2% in the first-line IST group (P = 0.033). Therefore, we believe that Haplo-HSCT can be a first-line treatment for paediatric SAA.

## Introduction

Aplastic anemia (AA) is a group of bone marrow failure diseases characterized by hemorrhage, anemia, infection, and hypoplasia of bone marrow and pancytopenia. According to the severity of AA, it can be classified as non-severe AA (NSAA), severe AA (SAA), and very severe AA (VSAA) 1. With the prolongation of the disease course, the majority of children with NSAA may progress to SAA, while due to the rapid onset and progression of SAA, children mainly die of complications related to severe infection and intracranial hemorrhage, with a high fatality rate. The first-line treatment for AA includes immunosuppressive therapy (IST) and hematopoietic stem cell transplantation (HSCT). IST consists of high-dose cyclophosphamide, antithymocyte globulin (ATG), etc. The disadvantages of IST mainly include a long reaction time of hematology during treatment, easy relapse, and an increased risk of clonal evolution 2-4. HSCT is the only effective radical therapy for children with SAA 1, including matched related donor-HSCT (MRD-HSCT), matched unrelated donor-HSCT (MUD-HSCT), haploidentical-HSCT (Haplo-HSCT), etc. 5. At present, MRD-HSCT and MUD-HSCT are the preferred methods of transplantation. However, due to Chinese one-child policy and limited bone marrow bank resources, only few children can find eligible donors. In addition, the matching of MUD takes a long time, while several children with critical disorder or severe infection mainly do not have enough time to wait for the completion of matching. Therefore, Haplo-HSCT, which is easily available to donors and has a short matching time, has gradually become one of the important treatment options for SAA patients. In recent years, with the joint efforts of domestic and foreign scholars, Haplo-HSCT has made a great progress 6. A large number of studies have shown that Haplo-HSCT has a remarkable efficacy in the treatment of children with SAA compared with matched HSCT 7-10. The present study aimed to summarize the latest data from patients with SAA who underwent HSCT in our hospital from June 2006 to January 2021, and to compare them with those who received IST in our hospital during the same period, providing evidence for the feasibility and effectiveness of Haplo-HSCT in the early-stage of SAA.

## Methods

### Patients

In total, 210 patients who were diagnosed with SAA in our hospital from June 2006 to January 2021 were enrolled in the study. There were 147 cases in first-line IST group and 63 cases in first-line HSCT group. All cases were diagnosed according to Chinese Expert Consensus on Diagnosis and Treatment of Aplastic Anemia (2022) 11, and were classified on the basis of the Camitta's criteria 12.

After admission, routine blood test, bone marrow biopsy, lymphocyte subset, chromosome karyotype analysis, and fusion gene test were conducted, and relevant examinations for connective tissue disease and paroxysmal nocturnal hemoglobinuria were performed for definite diagnosis; liver and kidney functions, Epstein-Bar virus (EBV), cytomegalovirus (CMV), human parvovirus B19 (HPV-B19), and infectious diseases were tested to evaluate patients' general conditions. In addition, all patients underwent chromosome breakage test and gene sequencing analysis to rule out the possibility of inherited bone marrow failure syndromes, whether they were accompanied with characteristic somatic manifestations or not.

The inclusion criteria were as follows: ① Meet the SAA diagnostic criteria and severity classification (mentioned above); ② Patients aged ≤ 18 years old; ③ Patients and their guardians who were fully aware of the clinical efficacy and prognosis of HSCT and IST for treatment of SAA, and signed the written informed consent form. ④ Previous HSCT results have been unsatisfactory, and the patient and their parents hope for a second transplant. The exclusion criteria were as follows: ① Not meeting the selection criteria. ② Irregular medication use and failure to follow up as required, which affects the evaluation of therapeutic effects. ③ Incomplete data affects the judgment of efficacy and safety.

### First-line HSCT group

#### Patients' data

The inclusion criteria were as follows: ① The patient declined IST; ② Transfusion dependence; ③ Eligible donors and appropriate conditions for HSCT.

A total of 63 patients with SAA were enrolled, including 32 (50.8%) male and 31 (49.2%) female patients; the patients' median age was 73 (range, 13-164) months. The median time from diagnosis to transplantation was 2 (range, 1-26) months; 36 (57.1%) cases received Haplo-HSCT, and 27 (42.9%) cases underwent MRD-HSCT. Besides, 27(42.9%) cases were of the same sex as donors; 36 (57.1%) cases had the same blood group. The median follow-up time was 58.5 (range, 2-179) months.

#### Conditioning regimens

The basic conditioning regimen included 50 mg/kg cyclophosphamide (Cy) and 3 mg/kg ATG at 2-5 days prior to transplantation. In addition to the basic regimen, 56 patients were given 25 mg/m^2^ Fludarabine at 2-6 days before transplantation to enhance immunosuppressive effects. Before the administration of Cy, all patients were adequately hydrated and alkalized, and mesna was given to prevent hemorrhagic cystitis.

#### Mobilization and collection of hematopoietic stem cells

The recombinant human granulocyte colony stimulating factor (G-CSF, 5 μg/(kg • d)) was subcutaneously injected for hematopoietic stem cell mobilization at 4-6 days prior to transplantation, and it was divided into 2 doses, once every 12 h for 4-6 days. During this period, routine blood test was performed every day. On the day of transplantation, peripheral blood stem cells were collected by a blood cell separator. After that, the number of mononuclear cells and CD34^+^ cells were counted by flow cytometry, and they were then cryopreserved. If the number of cells collected at the first time fails to meet the criteria for purification and engraftment, that's, the number of mononuclear cells is ≥ 5.0 × 10^8^/kg and that of CD34^+^ cells is ≥ 2 × 10^6^/kg, the peripheral blood hematopoietic stem cells can be collected for additional 1-2 times.

#### Diagnosis and prevention of graft-versus-host disease (GVHD)

Acute GVHD (aGVHD) and chronic GVHD (cGVHD) were evaluated by the modified Glucksberg grading system 13 and Chinese Consensus on the Diagnosis and Management of Chronic Graft-Versus-Host Disease (2021) 14, respectively. The prevention regimen for GVHD is based on the transplantation type. Here, 19 MRD cases, and 3 Haplo cases only received cyclosporine A (CsA); 8 MRD cases and 13 Haplo cases received CsA + short-term methotrexate (MTX); 20 Haplo cases received CsA + MTX + mycophenolate mofetil.

#### Prevention and treatment of infections

Compound sulfamethoxazole was given to prevent pneumocystis jiroveci pneumonia before conditioning regimen. Those who received broad-spectrum antibiotics also received imidazole antifungals to prevent fungal infections. Acyclovir was prescribed to prevent viral infection, and viral DNA copies were monitored weekly. Strict reverse isolation measures were implemented in the transplantation chamber. Routine disinfection of skin and mucous membrane, as well as perianal and catheter care were carried out every day until the patient left the transplant chamber.

Once bacterial infection occurs before stable stem cell engraftment, blood culture or infection site culture and drug sensitivity test shall be performed as soon as possible, and broad-spectrum antibiotic empirical treatment is simultaneously provided, followed by adjustment according to pathobiology assay. If cytomegalovirus infection occurs, antiviral drugs, such as ganciclovir or sodium phosphonoformate hexahydrate, were given until the viral DNA copies return to normal.

#### Engraft Monitoring

Routine blood test was performed daily after transfusion. The day of neutrophil engraftment was defined as the first day of 3 consecutive days, in which the absolute neutrophil count (ANC) was ≥ 0.5 × 10^9^/L, and the day of platelet engraftment was the first day of 7 consecutive days without transfusion in which the platelet count (PLT) was ≥ 20 × 10^9^/L 15. If ANC is < 0.5 × 10^9^/L at 28th day, primary engraftment failure is expected. Secondary engraftment failure was defined as a recurrent ANC <0.5x10^9^/L for at least 7 days 16.

#### Efficacy evaluation

Main outcome measures included engraftment rate and hematopoietic reconstitution time, the incidence of GVHD, infection rate after transplantation, 3- and 5-year survival rates, failure-free survival (FFS) rate, etc. Survival was defined as the time between transplantation and death from any cause or time of the last follow-up. FFS was defined as the day from transplantation to the occurrence of the first event (including death from any cause, non-response, recurrence) or from transplantation to the last follow-up 17.

### First-line IST group

#### Patients' data

The inclusion criteria were as follows: ① No definite active infection and/or massive bleeding at the start of treatment; ② No allergic reaction to all drugs administered for treatment; ③ No hepatic and renal insufficiency, malignancy, cognitive impairment or psychiatric disorders.

147 patients with SAA were enrolled, including 81 (55.1%) male and 66 (44.9%) female patients. The patients' median age was 70 (range, 18-201) months. The median time from diagnosis to IST was 0 (range, 0-11) month. Besides, 14 (9.5%) cases received ATG only; 37 (25.2%) cases received ATG + TPO; 96 (65.3%) cases received ATG + eltrombopag. The median follow-up time was 47 (range, 2-167) months.

#### Treatment regimen

1. ATG regimen: ATG (3.5 mg/(kg • d)) was intravenously administrated for 5 consecutive days. On the first day, 2.5 mg ATG was added to 100 ml normal saline that was given intravenously for 1 h for biological test. If no allergic reaction occurred, administration of the remaining ATG was continued. The initial dose of CsA was 3 ~ 5 mg/(kg • d) in 2 divided doses, then, the dose was adjusted according to the concentration of CsA in plasma, and the trough concentration of CsA was maintained at 150 ~ 250 μg/L.
2. ATG combined TPO regimen: The dosage and application method of ATG and CsA were the same as before. At the end of 5 days of ATG infusion, recombinant human thrombopoietin was injected subcutaneously, 7500 U/dose for patients with body mass ≤ 30 kg and 15000 U/dose for patients with body mass> 30 kg every other day. Injection was stopped for 3 months or PLT was > 100x10^9^/L.
3. ATG + eltrombopag regimen: The dosage and application method of ATG and CsA were the same as before. Eltrombopag (25 or 50 mg/dose) was orally infused once a day. If there was no significant adverse reaction or PLT was <20 × 10^9^/L or was still dependent on platelet transfusion, the dose was elevated by 25 mg every 2 weeks to the maximum tolerated dose of 150 mg/day.

#### Post-treatment assessments

Evaluation criteria after initial treatment of SAA were as follows: ① Complete remission (CR): hemoglobin > 100 g/L, PLT > 100 × 10^9^/ L and ANC> 1.5 × 10^9^/ L; ② Partial remission (PR): Patients were no longer dependent on blood transfusion, the results of hematological examination were improved, and no longer met SAA standards, while routine blood test did not meet CR criteria; ③ No remission (NR): Patients were not removed from blood transfusion support treatment and/ or the hematological examination until still meeting SAA criteria. TD-NSAA post-treatment assessment criteria were as follows: ① CR: The same as mentioned above; ② PR: At least one hemocyte cell line reached normal level or doubled, or initial hemoglobin <60 g/L increased by at least 30 g/L after treatment, or initial ANC <0.5 × 10^9^/ L increased by at least 0.5 × 10^9^/L after treatment, or initial PLT <20 × 10^9^/ L increased by at least 20 × 10^9^/L after treatment; ③ NR: The results of routine blood test decreased or did not reach PR criteria. [18]CR + PR was the objective response rate (ORR).

#### Efficacy evaluation

Main outcome measures included 3- and 5-year survival rates, FFS rate, etc. Survival was defined as the time between transplantation and death from any cause or time of the last follow-up. FFS was defined as the day from transplantation to the last follow-up or treatment failure, including death, nonresponse within 6 months, relapse, disease progression requiring a second cycle of IST or salvage HSCT and clonal evolution. Recurrence was defined as transfusion dependence or ANC <0.5 × 10^9^/L. Clonal evolution was defined as the transition to paroxysmal nocturnal hemoglobinuria or myelodysplastic syndrome or acute myeloid leukemia 19. The follow-up was ended at October 31, 2023.

### Statistical analysis

SPSS 25.0 software (IBM, Armonk, NY, USA) was used for statistical analysis. The measurement data were expressed as mean ± standard deviation (x ± s) and Median (minimum value - maximum value). Categorical variables were compared by Chi-square test, independent two-samplet-test and non-parametric rank-sum test was used for comparing continuous variables. Survival rate in each group was analyzed using Kaplan-Meier method, and the log-rank test was used to assess whether there were differences in subgroup survival curves. P < 0.05 was considered statistically significant. The proportion of missing data is relatively small (<5%), and most of the missing data types are binary data. A multiple imputation method based on logistic regression was used to supplement the data. The maximum, minimum, interquartile value of the data was obtained by description analysis, the data can be seen whether there are extreme values in the data, and the data can be analyzed after the extreme values are excluded.

## Results

### Haplo vs. MRD

#### Patients' characteristics

A total of 63 patients were divided into Haplo group (N = 36) and MRD group (N = 27) according to the transplant type. All patients did not receive IST, some were in critical conditions or complicated with serious infections when transferred to our hospital for treatment, while parents of the remaining patients refused IST. Patients' characteristics are listed in Table 1. The age of patients in the Haplo group was younger than that in the MRD group. In order to promote engraftment and reduce rejection, for the majority of patients in the Haplo group, fludarabine was added to the conditioning regimens. Moreover, in the Haplo group, grafts were all BM+PB to facilitate engraftment, and more mononuclear cells and CD34+ cells were reinfused into patients.

#### Reconstruction of hematopoietic function

Hematopoietic reconstitution was successfully conducted in 58 patients. The median time of ANC engraftment and PLT engraftment was 12 (range, 10-20) and 14(range, 7-37) days in the Haplo group, and 11.5 (range, 10-18) and 12 (range, 8-30) days in the MRD group, respectively. There was no significant difference between the two groups (Table 2). Moreover, 3 patients failed in primary engraftment, all of whom were in the Haplo group. Secondary engraftment failure occurred in 2 patients, including 1 in the Haplo group and 1 in the MUD group.

#### GVHD

Grade Ⅰ-Ⅳ aGVHD occurred in 31 of 63 patients (46.0%). In the Haplo group, the incidence of aGVHD was 61.1%, which was significantly higher than that in the MRD group (25.9%, P = 0.006), but the majority of children were of grade Ⅰ with skin involvement. There was no significant difference in the incidence of cGVHD between the two groups (41.7% vs. 33.3%, P = 0.326) (Table 2).

#### Infection-related complications

A total of 29 patients developed CMV infection after transplantation, in which the incidence was 55.6% in the Haplo group and 33.3% in the MRD group, and the difference was not statistically significant (P = 0.080), as shown in Table 2. Among the 29 patients infected with CMV after transplantation, 25 cases developed Cytomegaloviremia, 3 cases developed CMV pneumonia, and 1 case developed CMV enteritis. There were 17 cases of EBV infection after transplantation, in which the incidence rate was 30.6% in the Haplo group and 22.2% in the MRD group. There was no significant difference between the two groups (P = 0.461), as shown in Table 2. Among the 17 patients infected with EBV after transplantation, 2 cases developed lymphoproliferative disease, and 1 case developed EBV encephalitis.

#### Survival analysis

**Figure 1:**
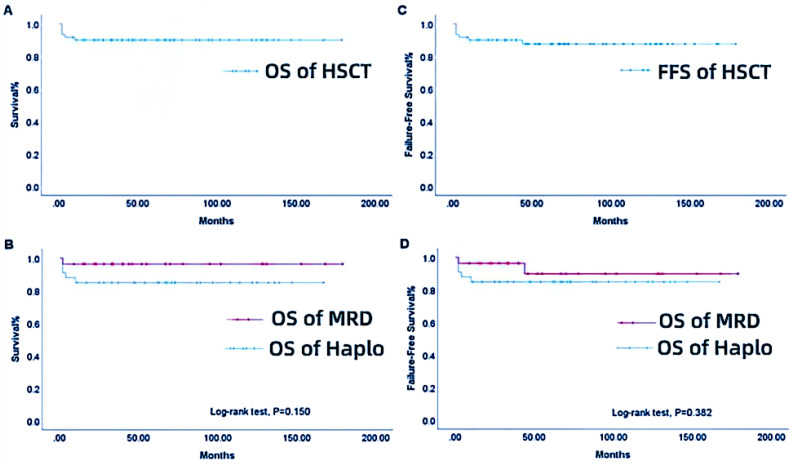
OS (A), group-based OS (B), overall FFS (C), and group-based FFS (D) for patients in the HSCT group from 2006 to 2021. MRD-HSCT, matched related donor or matched unrelated donor-hematopoietic stem cell transplantation; Haplo-HSCT, haploidentical-hematopoietic stem cell transplantation group.

The median follow-up time was 58.5 (range, 2-179) months, and the overall survival (OS) rate was 90.0%, as shown in Figure 1A. The 3- and 5-year cumulative survival rates were both 84.9% in the Haplo group and 96.3% in the MRD group, and the difference was no statistically significant (P = 0.150, Figure 1B). The overall FFS rate was 88.3%, as displayed in Figure 1C. The 3- and 5-year cumulative FFS rates were both 84.9% in the Haplo group and 92.6% in the MRD group, respectively, with no statistically significant difference (P = 0.382, Figure 1D). A total of 6 patients died, including 5 patients in the Haplo group, 1 patient died of after transplantation respiratory failure due to severe pneumonia before MRD transplantation. In the Haplo group, 4 patients died of post-transplant sepsis shock and Multiple organ failure, one of them has infection before transplantation. One patient died of severe infection. The infection occurred before the second transplantation, which was uncontrollable during the transplantation and continued to worsen until the patient died.

#### Health economics analysis

The total cost of transplantation between the Haplo and MRD groups was compared, and it was revealed that, the total cost of transplantation in the Haplo group was 252700.37±16517.41(RMB), while that was 218691.38±13503.26(RMB) in the MRD group (P = 0.127, Table 2).

### First-line IST vs. First-line Haplo-HSCT

#### General conditions

All patients in the IST group received first-line IST and were assigned to the first-line IST group (N=147), Besides, 36 patients in the Haplo group received first-line Haplo-HSCT (N = 36). The characteristics of patients in the two groups are shown in Table 3. Among patients who did not receive IST, some patients were in critical conditions or complicated with serious infections when they were transferred to our hospital for treatment and could not receive IST, while parents of other children refused to receive IST.

#### Survival Analysis

**Figure 2:**
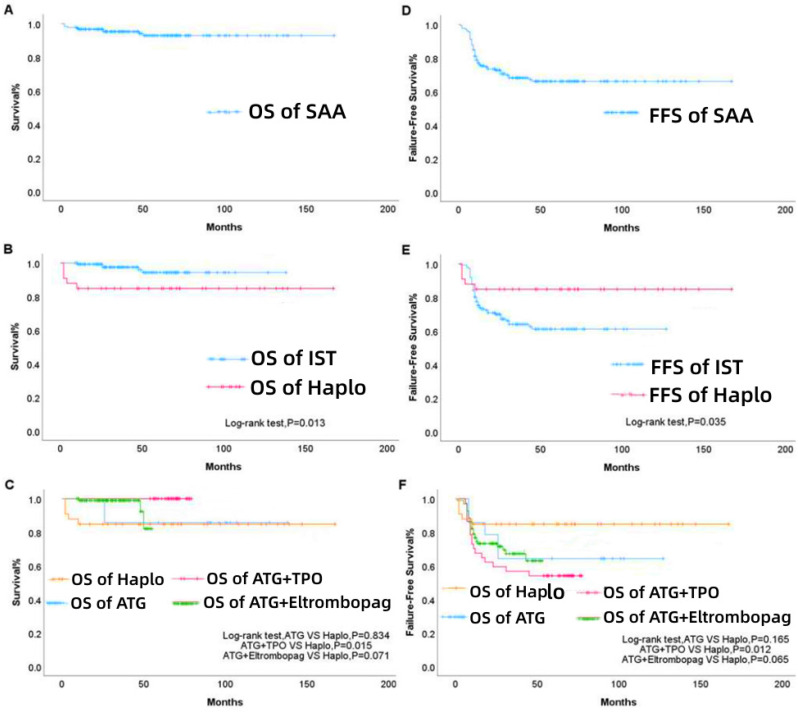
First-line treatment. OS (A), group-based OS (B), subgroup survival rate of IST (C), overall FFS (D), group-based FFS (E), and subgroup FFS rate of IST (F) for patients from 2006 to 2021. IST, immunosuppressive therapy; Haplo-HSCT, haploidentical-hematopoietic stem cell transplantation; ATG, antithymocyte globulin; TPO, thrombopoietin.

The median follow-up time was 47 (range, 2-167) months, and the OS rate was 94.4%, as illustrated in Figure 2A. The 3- and 5-year cumulative survival rates in the first-line Haplo group were both 84.9%, while those in the first-line IST group were 98.0% and 96.6%, respectively, with statistically significant difference (P=0.013, Figure 2B). According to the specific treatment strategy, the first-line IST group was further subdivided. The 3- and 5-year cumulative survival rates in the ATG group were both 85.7%, which were not significantly different from those of the first-line Haplo group (P = 0.834); the 3- and 5-year cumulative survival rates in the ATG + TPO group were both 100%, and the difference was statistically significant compared with the first-line Haplo group (P=0.015); the 3- and 5-year cumulative survival rates in the ATG+ eltrombopag group were 99.0% and 96.9%, respectively, indicating that there was no statistically significant difference compared with the first-line Haplo group (P=0.071, Figure 2C). The overall FFS rate was 68.9%, as shown in Figure 2D. The 3- and 5-year cumulative FFS rates in the first-line Haplo group were both 84.9%, while those were 66.7% and 65.3% in the first-line IST group, respectively, with statistically significant difference (P=0.013, Figure 2E). According to the specific treatment strategy, the first-line IST group was further subdivided. The 3- and 5-year cumulative FFS rates in the ATG group were both 64.3%, which were not significantly different from those of the first-line Haplo group (P = 0.165); the 3- and 5-year cumulative FFS rates in the ATG + TPO group were 56.8% and 54.1%, and the difference was statistically significant compared with the first-line Haplo group (P=0.012); the 3- and 5-year cumulative FFS rates in the ATG+ eltrombopag group were 70.8% and 69.8%, respectively, indicating that there was no statistically significant difference compared with the first-line Haplo group (P=0.065, Figure 2F). A total of 10 patients died; 5 patients were in the IST group, of whom 1 died of post-transplant infection and rejection, and 4 died of aplastic anemia and infection before transplantation; 5 patients were in the Haplo group, 4 patients died of post-transplant sepsis shock and Multiple organ failure, one of them has infection before transplantation. One patient died of severe infection; the infection occurred before the second transplantation, which was uncontrollable during the transplantation and continued to deteriorate until the patient died.

## Discussion

It is well known that AA is a rare disease characterized by two-line pancytopenia and bone marrow hematopoietic failure. The incidence rate of AA is (0.6-7)/1 million. The incidence of AA in children is slightly higher than that in other groups 20, and the degree is more serious. Among different types of AA, more than half of children have SAA 21. In case of late treatment of AA, the mortality rate can reach 80% within 2 years 22. With the improvement of supportive care, conditioning regimen and IST after transplantation, the OS rate of SAA patients treated with HSCT has been gradually improved.

At present, MRD-HSCT, as the first-choice therapy for SAA children, has a 5-year survival rate of about 80%, which reached 90% in young patients 23, 24. However, it is not easy to find eligible donors due to Chinese one-child policy, and about 70% of patients lack human leukocyte antigen (HLA)-identical sibling donors. Although the efficacy of MUD-HSCT is similar to MRD-HSCT 25, only few children can find a MUD donor. Furthermore, it takes up to several months to find an eligible donor, and patients requiring emergency transplantation encounter with limited time. Therefore, the first-line Haplo-HSCT has gradually attracted clinicians' attention. In addition, the 6-month response rate of IST is only 55-60% as the secondary therapeutic option. The 5-year recurrence rate is about 35% 26, and 5-6% of children may develop clonal transformation within 10 years 27, including myelodysplastic syndrome or acute myeloid leukemia. Studies shown that relapse and conversion to myelodysplastic syndrome and acute myeloid leukemia are more common after IST treatment, with a median recurrence rate of 33.5 months (5-228), the recurrence rate was 21.9%., twenty-six (5.5%) patients progressed to PNH, 20 (4.3%) progressed to MDS / AML, with a ten-year estimated overall survival (OS) of 80.9% ± 3%, among MRD-HSCT recipients, relapse rate of 4.9% with no clonal evolution and a 10-year OS of 94.5 ± 2%. 28 Moreover, due to the lack of horse ATG in China, rabbit ATG can only be used alternatively, while its efficacy is significantly lower than horse ATG 29, indicating the necessity of promoting first-line Haplo-HSCT in developing countries, such as China.

Donors for Haplo-HSCT are readily available and numerous patients have donors. Moreover, HSCT can be performed immediately, which is particularly important for critical patients who require prompt treatments. Haplo-HSCT can also be used to salvage transplant failure or to treat infection, and using the improvement of transplantation techniques, Haplo-HSCT has shown a better prognosis. Studies have shown that the engraftment rate and OS rate of Haplo-HSCT and MUD-HSCT were similar in children and adolescents 30, 31. Xu *et al.* 25 compared the prognosis of 158 SAA patients who received HSCT, and the results showed that in young patients, the OS and FFS rates of Haplo-HSCT and MUD-HSCT were similar. In Haplo-HSCT and MUD-HSCT groups, the 3-year survival rate was 86.1% and 91.3%, respectively (P =0.358), and the FFS rate was 85.0% and 89.8% (P=0.413), respectively. In the present study, Haplo-HSCT was recommended as an alternative therapy for MRD-HSCT. As for the source of graft, compared with bone marrow transplantation, peripheral blood hematopoietic stem cell transplantation has the advantages of rapid implantation, rapid immune reconstruction and low recurrence rate, which has been more and more widely used in clinical practice. However, some studies have found that peripheral blood hematopoietic stem cell transplantation leads to an increased incidence of GVHD. Meta-analysis has shown that peripheral blood hematopoietic stem cell transplantation in the treatment of hematological malignancies has a relatively low recurrence rate and a high survival rate, but it also has a high risk of A and cGVHD^32^. In this study, we mobilized bone marrow and peripheral blood by G-CSF and performed combined transplantation to promote Haplo-HSCT implantation and immune reconstruction and control the risk of GVHD. The MRD group still used routine peripheral blood transplantation. In addition, different from previous studies that used TBI and *in vitro* T cell removal, in this study, we also comprehensively improved the Haplo-HSCT protocol by adding fludarabine to replace T removal *in vitro* with T removal *in vivo*, and injecting sufficient MNC and CD34+ cells, and achieved good results. There was no statistically significant difference in hematopoietic reconstitution time, cGVHD rate, CMV, EBV infection rates, OS rate and FFS rate between the Haplo and MRD groups, the long-term survival of children was confirmed.

AA is a non-neoplastic hematologic disease, thus, avoiding acute or chronic GVHD after successful transplantation is the key to treatment. Although the incidence of aGVHD in the Haplo group was higher than that in the MRD group, the majority of children were of grade Ⅰ with skin involvement. There was no significant difference in the incidence of cGVHD between the two groups, which was related to the use of ATG for the prophylaxis of GvHD by *in vivo* T-cell depletion, low-dose fludarabine conditioning regimen, and GVHD prevention regimen with CsA + MTX + mycophenolate mofetil. In addition to GVHD, post-transplant viral infections pose a serious threat to patients. The present study showed that there was no significant difference in CMV and EBV infection rates between the two groups, which might be attributed to the active prophylactic use of antiviral drugs before and after transplantation, and sufficient MNC and CD34^+^ cells to facilitate the engraftment. Compared with the MRD-HSCT, there was an increased engraft failure rate and a higher incidence of graft dysfunction, thus, stem cell mobilization strategies, conditioning regimens, and graft selection need further exploration. Several studies have shown that there was no significant difference between Haplo-HSCT and MRD-HSCT in terms of hematopoietic reconstruction and overall prognosis 30, 33. Haplo-HSCT can be a first-line alternative for SAA children.

Compared with IST, Haplo-HSCT possesses the advantages of a high complete remission rate, low relapse and clonal transformation rates, and easy access to donors. In a retrospective study comparing the efficacy and safety of Haplo-HSCT and IST for SAA patients, 18 patients were treated with Haplo and 10 patients were treated with IST. The median follow-up time was 23.5 (range, 3-52) months. There was no significant difference in OS between the two regimens (P > 0.05) 34. In another study, 29 patients received IST and 20 patients underwent Haplo-HSCT, and there was no significant difference in 3-year survival rate between the two groups (P = 0.740), while FFS in the Haplo-HSCT group was significantly higher than that in the IST group (P = 0.043) 19. In the present study,147 patients received first-line IST and 36 patients underwent first-line Haplo-HSCT. FFS in the Haplo group was significantly higher than that in the IST group, and the quality of life was well, whereas OS rate was lower than that in the IST group. Therefore, the IST group was divided into ATG, ATG + TPO, ATG + eltrombopag groups, and compared with the first-line Haplo group. The results showed that only the ATG + TPO group had a higher OS rate than the Haplo group, and there was no significant difference between the other two groups. Among 37 patients in the ATG+TPO group, 17 patients failed at IST, presenting as transfusion dependence, of whom 7 patients underwent HSCT and 10 patients continued IST with the change of regimen. The OS rate was 100%, which may be associated with the higher failure rate of IST treatment and rapid transfer to transplantation or the use of other treatment regimens. Besides, the number of cases was limited, and there might be a certain contingency.

The results of the present study showed that the overall prognosis of the first-line Haplo-HSCT group was basically similar to that of the first-line IST group. However, it is necessary to consider the limitations of IST, such as high recurrence rate, low remission rate after recurrence, high probability of clonal transformation, in the IST group, some children missed the opportunity of transplantation and died of AA and infection before transplantation, children's life expectancy was longer, and the treatment aimed at cure rather than improvement. Therefore, Haplo-HSCT may be considered as a viable alternative in the absence of MRD donors.

This study is a single center retrospective study, there are still some limitations, including relying on existing clinical data and unable to control for other possible confounding factors, such as baseline characteristics of patients and the impact of other treatment measures, incomplete data due to retrospective study, small number of patients in some groups, baseline differences between groups, the possibility of underestimation of IST in the absence of horse ATG, etc. This study strengthens data quality control and validation, strengthens follow-up, as far as possible ensures data accuracy and completeness, and uses appropriate statistical methods for data processing, and in the future, we plan to conduct multicenter retrospective and prospective studies to further validate the conclusions. However, our data suggested Haplo-HSCT as a first-line treatment for pediatric SAA.

## Figures and Tables

**Table 1:** Patients' Characteristics (Haplo group vs. MRD group)

Variables	Haplo (N=36)	MRD(N=27)	P
Age (months), median (range)	51 (13-164)	95(17-156)	0.002
Male/female, no.	19/17	13/14	0.716
SAA/vSAA, no.	22/14	13/14	0.306
Neutrophil count (×109/L, mean ± SD)	0.39±0.11	0.55±0.12	0.337
Platelet count (×109/L, mean ± SD)	15.84±3.72	17.30±3.85	0.791
Interval between AA diagnosis and HSCT (months)	2 (1-9)	1 (1-26)	0.028
ABO matched, no. (%)	22(61.1%)	14(51.9%)	0.462
Donor-patient sex match, no. (%)	19(52.8%)	8(29.6%)	0.066
Graft type, no. (BM+PB/PB)	36/0	2/25	<0.001
MNCs infusion (×108/kg, mean ± SD)	17.97±1.79	12.38±1.13	0.011
CD34+ infusion (×106/kg, mean ± SD)	12.06±1.34	7.52±0.59	0.003
Conditioning regimen (FCA/CA)	33/3	12/15	<0.001

**Table 2:** Hematopoietic reconstruction, infection-related complications, and health economics after transplantation

	Haplo (N=36)	MRD(N=27)	P
Neutrophil engraftment, median (months, range)	12 (10-20)	11.5 (10-18)	0.141
Platelet engraftment, median (months, range)	14 (7-37)	12(8-30)	0.173
aGVHD, no. (%)	22 (61.11%)	7 (25.93%)	0.006
GradeⅡ-Ⅳ, no. (%)	9 (25.00%)	0 (0%)	0.147
cGVHD, no. (%)	15 (41.67%)	8(29.63%)	0.326
CMV, no. (%)	20 (55.56%)	9 (33.33%)	0.080
EBV, no. (%)	11 (30.56%)	6 (22.22%)	0.461
The total cost (RMB)	252700.37±16517.41	218691.38±13503.26	0.127

**Table 3:** Patients' Characteristics (first-line IST vs. first-line Haplo-HSCT)

	First-line IST (N=147)	First-line Haplo-HSCT (N=36)	P
Age(months), median (range)	70 (18-201)	51 (13-164)	0.003
Male/Female, no.	81/66	19/17	0.802
NSAA/SAA, no.	76/71	0/36	0.310
Interval between AA diagnosis and SCT/IST (month)	0(0-11)	2 (1-9)	<0.001
